# Improved one-tube RT-PCR method for simultaneous detection and genotyping of duck hepatitis A virus subtypes 1 and 3

**DOI:** 10.1371/journal.pone.0219750

**Published:** 2019-08-01

**Authors:** Xueming Chen, Yuhuan Chen, Chungguo Liu, Xiaojun Li, Hongyu Liu, Xiuchen Yin, Xiaofei Bai, Ming Ge, Hongyan Chen, Ming Liu, Yuanzhao Du, Gencheng Fan, Yun Zhang

**Affiliations:** 1 State Key Laboratory of Veterinary Biotechnology, Harbin Veterinary Research Institute of Chinese Academy of Agricultural Sciences, Harbin, China; 2 College of Veterinary Medicine, Northeast Agriculture University, Harbin, China; 3 State Key Lab of Genetically Engineered Veterinary Vaccine, YeBio of Qingdao, Qingdao, China; University of Valencia, SPAIN

## Abstract

**Background:**

The cocirculation of duck hepatitis A virus subtypes 1 (DHAV-1) and 3 (DHAV-3) in ducklings has resulted in significant economic losses. Ducklings with DHAV-1 or DHAV-3 infection show similar clinical signs and gross lesions; hence, it is important to identify the viral subtypes in infected ducklings as early as possible for better clinical management.

**Methods and results:**

Based on multiple 5’ noncoding region (5’-NCR) sequences of DHAV-1 and DHAV-3 strain alignments, universal and type-specific primers were designed and synthesized. With three primers in one-tube reverse transcription-PCR (RT-PCR), reference DHAV-1 and DHAV-3 isolates ranging over 60 years and across many different countries were successfully amplified, indicating that the primer sequences were completely conserved. The sequence results and the sizes of amplicons from reference DHAV-1 and DHAV-3 isolates are completely correlated with their subtypes. Moreover, with this one-tube RT-PCR system, amplicon sizes from liver samples of reference DHAV-1- or DHAV-3-infected birds fit closely with their subtypes, which was determined by virus isolation and neutralization testing. No other duck-origin RNA viruses were detected. The sensitivity of viral RNA detection was 10 pg. With this system, 20% subtype 1, 45% subtype 3, and 9% coinfection of two subtypes were detected in 55 clinical samples.

**Conclusions and significance:**

This novel approach could be used for rapidly typing DHAV-1 or DHAV-3 infection in routine clinical surveillance or epidemiological screening.

## Introduction

Duck hepatitis A virus (DHAV) is an acute, highly lethal and contagious etiological agent of viral hepatitis of ducklings [1, 2]. Based on phylogenetic analyses, DHAV has been classified into 3 genotypes: DHAV type 1 (DHAV-1), type 2 (DHAV-2), and type 3 (DHAV-3). DHAV-2 and DHAV-3 are genetically and serologically different from DHAV-1[3–6]. The most common, virulent, and worldwide distribution subype is DHAV-1. DHAV subtype 2 is only limited in Taiwan. DHAV subtype 3 is often prevalent in South Korea, Vietnam, and China [3–11]. DHAV-1 occurs worldwide and threatens all duck-growing farms, causing more than 80% mortality in ducklings.

DHAV is a member of *Avihepatovirus* in the family *Picornaviridae* [4, 12, 13]. The genomes of DHAV-1, DHAV-2 and DHAV-3 are single-stranded, positive-sense RNA containing a single, large open reading frame (ORF), which encodes a polyprotein measuring 2249 aa flanked by the 5’- and 3’-noncoding region (NCR). The genomes of DHAV-1 and DHAV-3 are organized as members of the family *Picornaviridae*: 5^’^NCR-VP0–VP3–VP1–2A–2B–2C–3A–3B–3C–3D–3^’^NCR. There are several approaches for the diagnosis of duck hepatitis disease, including examining clinical signs, determining gross pathological changes, and reproducing the disease in susceptible ducklings. However, these methods have been shown to be incapable of detecting or discriminating DHAV-1 and DHAV-3 because of their similar clinical symptoms and pathology [14]. The neutralization test [15], virus isolation combined with PCR [16], and immunofluorescence assays [11, 17] are reliable for typing DHAV, but they are labor-intensive and time-consuming. Enzyme-linked immunosorbent assays (ELISA) are reliable test for detecting DHAV-1/DHAV-3, but antigen preparation in ELISA is also cumbersome [1,18].

To date, RT-PCR has been used widely to simultaneously detect infections of animal and plant viruses. Due to the emergence of new DHAV-3 subtypes, the widely preferred method is molecular genotyping [8, 10, 19–24], in which the subtype of an isolate is determined largely by sequencing part of the viral genome and then using phylogenetic analysis to compare it with already known subtype reference sequences. Some RT-PCR approaches are employed for the detection and genotyping of DHAV-1 and DHAV-3, but the amplicons are difficult to discriminate by size, and this approach must also employ sequencing results analysis [8]. Moreover, this approach is time-consuming, labor-intensive, and expensive. Duplex PCR differential approach for DHAV-1 and DHAV-3 was successfully developed that enabled easy discrimination of amplicons by size, but multiple sets of primers require multiple rounds of PCR [10, 21, 22], which are also labor-intensive, expensive and inconvenient. The third real-time PCR detection approach [19, 24] is accurate, but this assay is cumbersome, labor-intensive, and expensive and requires special equipment. Therefore, it may be desirable to develop a rapid, simple, sensitive, and economical diagnostic assay for genotyping DHAV-1 and DHAV-3.

Previously, the polyprotein genome sequence has usually been used to judge phylogenetic relationships within picornavirus genotypes, which correlated with virus serotypes or subtypes. In this study, we conducted sequence analysis based on the available sequence information of DHAV-1 and DHAV-3 in GenBank. Phylogenetic analysis indicated that the 5’-NCR could be used as a target gene for DHAV-1 and DHAV-3 subtype analysis. Using universal primer and type-specific primers targeted to the 5’-NCR, we developed a one-tube RT-PCR method for the simultaneous detection and genotyping of DHAV-1 and DHAV-3 without amplicon sequencing. With this one-tube RT-PCR system, we demonstrated that the sequence results and the size of amplicons of reference viruses or samples of reference virus-infected birds fit well with the gold standard method: virus isolation and neutralization test (VI/NT). Fifty-five clinical samples were successfully screened with this one-tube RT-PCR system. Since DHAV-2 is unavailable in China, we examined DHAV-1 and DHAV-3 detection in this study.

## Materials and methods

### Ethics statement

All experiments involving animals were approved by the Animal Welfare and Ethical Censor Committee at Harbin Veterinary Research Institute (HVRI) and Animal Ethics Committee of the HVRI of the Chinese Academy of Agricultural Sciences with license SYXK (Heilongjiang) 2011022.

### Viruses

Three DHAV-1 strains, DRL-62 (from ATCC), R85952 (from ATCC), and HP-1 [1, 25], five DHAV-3 strains, JT and GY [14], and three recently isolated strains, HLJ-1, ZJ-01309, and SD0517, were used as references in this study. The viruses were propagated in 12-day-old embryonated duck eggs (free of DHAV-1 and DHAV-3 infections) as described previously [14], and the allantoic fluids of infected eggs were collected and stored at -80°C. Avian influenza viruses (AIV) WY11 and WY24 [26], Newcastle disease virus (NDV) vaccine strain Lasota, Muscovy duck reovirus (DRV) S14 strain [27], and duck Tembusu virus (DTMUV)TA strain [28] were used for PCR specificity analysis.

### Clinical samples

Fifty-five clinical liver samples with hemorrhagic lesions were collected and used to screen DHAV-1 and DHAV-3 field infections in bird flocks from 2015 to 2017 in China.

### Phylogenetic analysis of the 5’-NCR and the polyprotein of DHAV-1 and DHAV-3

Sixteen nucleotide sequences from the 5’-NCR and the polyprotein of DHAV-1 and DHAV-3 in GenBank were used to create phylogenetic trees for relationship studies. The strain information from GenBank includes nine reference strains of DHAV-1 and seven of DHAV-3, which covered the greatest variety of countries and ranged most widely in time (across 60 years) (Table 1). LASERGENE 7.1 software (DNASTAR, 6.0, Madison, WI, USA) was used for sequence analysis. Phylogenetic trees were generated by using the Neighbor-Joining method in MEGA 4.0 software [29]; Bootstrap probabilities were calculated with 1000 replicates. The phylogenetic trees were visualized by using the program TreeView.

**Table 1:** Virus information used for phylogenetic analysis in this study.

DHAV type	Isolates	GenBank No	Country	Time
DHAV-1	DRL-62	DQ219396	USA	1970
	R85952	DQ226541	USA	1955
	DHV-HS	DQ812094	Korea	1994
	C80	DQ864514	China	2011
	HP-1	EF151312	China	1975
	5886	DQ249301	USA	2005
	03D	DQ249299	Taiwan	2003
	H	JQ301467	England	1993
	DHV-HSS	DQ812092	South Korea	1995
	A66	DQ886445	China	2006
	JX	EF093502	China	2006
	E53	EF151313	China	2006
	S	EF417871	China	2007
	R	EF585200	China	2007
	ZJ	EF382778	China	2008
	F	EU264072	China	2007
DHAV-3	AP-03337	DQ256132	Korea	2003
	AP-04114	DQ812093	Korea	2006
	AP04203	DQ256134	Korea	2007
	C-GY	EU352805	China	2007
	JT	JF835025	China	2010
	SD01	GQ485310	China	2008
	SD02	GQ485311	China	2008
	JS2010	HQ654774	China	2010
	AP-04009	DQ256133	South Korea	2011
	B63	EU747874	China	2011
	DN2	JF914944	Viet Nam	2009
	LS	KP233203	China	2014

### Primers

The 5’-NCR was selected for primer design based on GenBank sequence alignments of DHAV-1 and/or DHAV-3 (Table 1) by using the CLUSTAL W computer program (DNASTAR 6.0, Madison, WI, USA). The forward primer pAF was designed for the detection of both DHAV-1 and DHAV-3 subtypes based on conserved regions for both DHAV-1 and DHAV-3. The subtype-specific reverse primers pA1R and pA3R were designed based on conserved subtype-specific regions for DHAV-1 or DHAV-3, respectively. A PCR assay using these three primers in one tube was evaluated for detection and genotyping DHAV-1 and DHAV-3.

### RNA extractions and one-tube/one-step RT-PCR

For this experiment, 200 μl of reference virus stock (including three DHAV-1 related viruses, DRL-62, R85952, and HP-1; and five DHAV-3 related viruses, JT, GY, HLJ-1, ZJ-01309, and SD0517) or supernatants of clinical sample homogenates were used for RNA extraction with TRIzol reagent (Invitrogen, Life Technologies, Carlsbad, CA) according to the manufacturer’s instructions. The RNA was eluted in 20 μL of DEPC water. One-Step RT-PCR (Qiagen, Hilden, Germany) was carried out in a 25-μL reaction volume containing 5 μL of 5×PCR buffer, 1 mM dNTP mix, 2 μL of extracted RNA, 2 μL of pAF and 1 μL of each pA1R/pA3R (primer concentration 2.5 pmol/μl), 2 μL of One-Step RT-PCR Enzyme Mix. A thermal cycler (Biometra, Germany) was used for the RT-PCR. The RT-PCR mixture was subjected to the following thermal cycle conditions: a reverse transcription step at 50°C for 30 min, a denaturation step at 95°C for 15 min followed by 15 cycles of 95°C for 30 s, 55°C for 30 s and 72°C for 30 s; 30 cycles of 95°C for 30 s, 60°C for 30 s, 72°C for 30 s; and a final extension for 3 min at 72°C. The PCR products were detected by 2% agarose gel electrophoresis (Sigma–Aldrich, St. Louis, MO, USA) stained with ethidium bromide. The appropriate sizes of PCR products were extracted using an Agarose Gel DNA extraction kit (Watson Biotechnologies, Inc. Shanghai, China) and then submitted for sequencing. To exclude laboratory contamination, RNA extractions and PCR mixture preparations were processed with different sets of pipettes and filter tips. Each RT-PCR was used to screen for contamination using negative reagent controls.

### Cloning and sequence analysis

Appropriate sizes of PCR products were purified and cloned into a pMD18-T cloning kit (TaKaRa Biotechnology Co., Ltd.) as described previously (27). Positive plasmids were purified using a QIAprep spin miniprep kit (Qiagen, Valencia, CA, USA) and submitted for sequencing (DNA Sequence Service, TaKaRa Biotech Co). The nucleotide sequences were analyzed with the MegAlign program and deposited in GenBank.

### Sensitivity of the RT-PCR assay

The sensitivity of the one-tube RT-PCR for detecting DHAV-1 and DHAV-3 was measured by using 10-fold serial dilutions of the allantoic fluids of the HP-1 or JT strains. RNA from each dilution (10-fold, 10^−1^ to 10^−7^) of viruses was extracted as described above. Two microliters of RNA (approximately 10^2^ to 10^−4^ ng) from each dilution in a 25-μL reaction volume was employed with one-tube RT-PCR. Sensitivity for mixed stock (mixed HP-1 and JT) was also evaluated. Briefly, each individual 10-fold serial dilution of HP-1 and JT was mixed prior to RNA extraction. The sensitivity of the RT-PCR process was evaluated as described above.

### One-tube RT-PCR for DHAV-1/-3 detection samples from experimentally infected birds

To evaluate the efficiency of the established RT-PCR for detection samples from infected birds, ducklings were infected with reference DHAV-1/-3. Briefly, the **e**leven groups of birds (each group consisted of 10 1-day-old SPF ducklings) were intramuscularly inoculated with 10^4.5^ duck embryo lethal dose (ELD_50_) of single DHAV-1 (DRL-62, R85952, and HP-1), single DHAV-3 (JT, GY, HLJ-1, ZJ-01309, and SD0517), mixed stocks A (HP-1 and JT) (with equal dose) and B (DRL-62 and GY), and 0.2 ml PBS as a negative control. The birds were observed daily for clinical signs, gross lesions, and death. Liver samples from dead infected or noninfected control birds were collected for one-tube RT-PCR analysis and virus isolation combined with the neutralization test (VI/NT). Liver samples were pretreated before being subjected to RNA extraction or virus isolation as described previously (13, 29). Briefly, after three freeze–thaw cycles, liver samples were homogenized at 1:2 (v/v) in phosphate-buffered saline (PBS) and pelleted by centrifugation at 5,000×g for 15 min. The supernatants were collected for RNA extraction or VI/NT. Extracted RNA was then used for one-tube RT-PCR as described above. Amplicons of 214 bp and/or 289 bp were submitted for sequencing.

### Virus isolation and neutralization test (VI/NT) for DHAV-1/-3 detection

When supernatants of liver samples were determined with single amplicons of 214 bp or 289 bp by RT-PCR, we next conducted virus isolation from these positive supernatants. Briefly, 0.2 mL selected positive homogenized supernatants were injected into 11-day-old SPF duck embryos. Allantoic fluids were collected after the embryos died over 24 h of inoculation and stored at -70°C until use. DHAV in the allantoic fluids was then characterized by a neutralization test using anti-DHAV-1 or anti-DHAV-3 sera [1, 14]. Virus neutralization tests were performed using the constant virus variable serum method as described previously [1]. Sera against DHAV-1 and DHAV-3 were inactivated at 56°C for 30 min before the neutralization test. SPF duck embryos were used as the indicator in the virus neutralization test. The endpoint titer of the serum against homologous and heterologous viruses was calculated by the method of Reed and Muench [30]. Antigenic relationships (r-values) were calculated as the ratio between heterologous/homologous serum titer. Samples of DHAV-1 and DHAV-3 mixed-infection birds were not subjected to VI/NT.

## Results

### Phylogenetic analysis of the 5’-NCR and the polyprotein genome of DHAV-1 and DHAV-3

**Fig 1:**
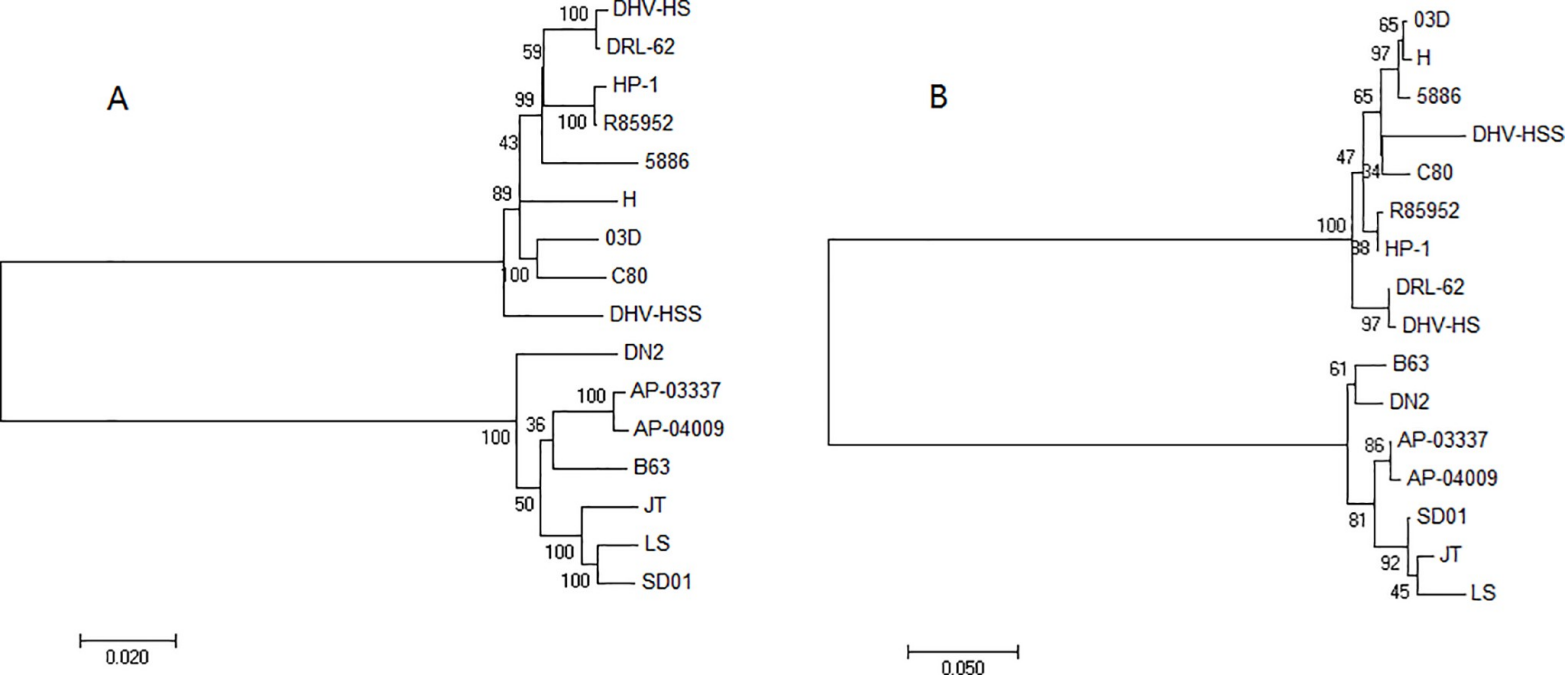
Phylogenetic analysis of the polyprotein genome (A) and the 5’-NCR (B) of the DHAV-1 and DHAV-3 strains from GenBank. The numbers above and below the branches indicate bootstrap values. The scale bar represents the nucleotide substitutions per site.

The polyprotein genome sequence is usually used to judge phylogenetic relationships within picornavirus genotypes, which correlate with virus serotypes. To prove that the 5’-NCR sequence could be used for virus genotyping, we compared phylogenetic trees constructed by polyprotein and the 5’-NCR. The phylogenetic tree constructed by the polyprotein genome clearly demonstrated that DHAV-1 or DHAV-3 constitute two monophyletic clades, DHAV-1 clade (9 strains) and DHAV-3 clade (7 strains) (Fig 1A). Similar to the phylogenetic tree of the polyprotein, the phylogenetic tree built by the 5’-NCR also forms two groups: 9 strains of DHAV-1 form group A and 7 strains of DHAV-3 form group B (Fig 1B). Sequence analysis indicated that the polyprotein genome of DHAV-1 and DHAV-3 strains showed 93.8–100% homology within the same genotype (clade) and 66.3–70.2% identity between two genotypes (clades). Similar to that of the polyprotein genome, the 5’-NCR of DHAV-1 or DHAV-3 strains from different locales and years were closely related within the same genotype (clade), showing 94.2–99.8% homology within genotypes and 51.4–66.7% identity between different genotypes (clades). The similar pattern of phylogenetic trees and the similar genetic distance of the 5’-NCR and the polyprotein genome of DHAV-1 and DHAV-3 suggested that the 5’-NCR might also be used to study genotyping or phylogenetic relationships.

### Primer sequences

**Fig 2:**
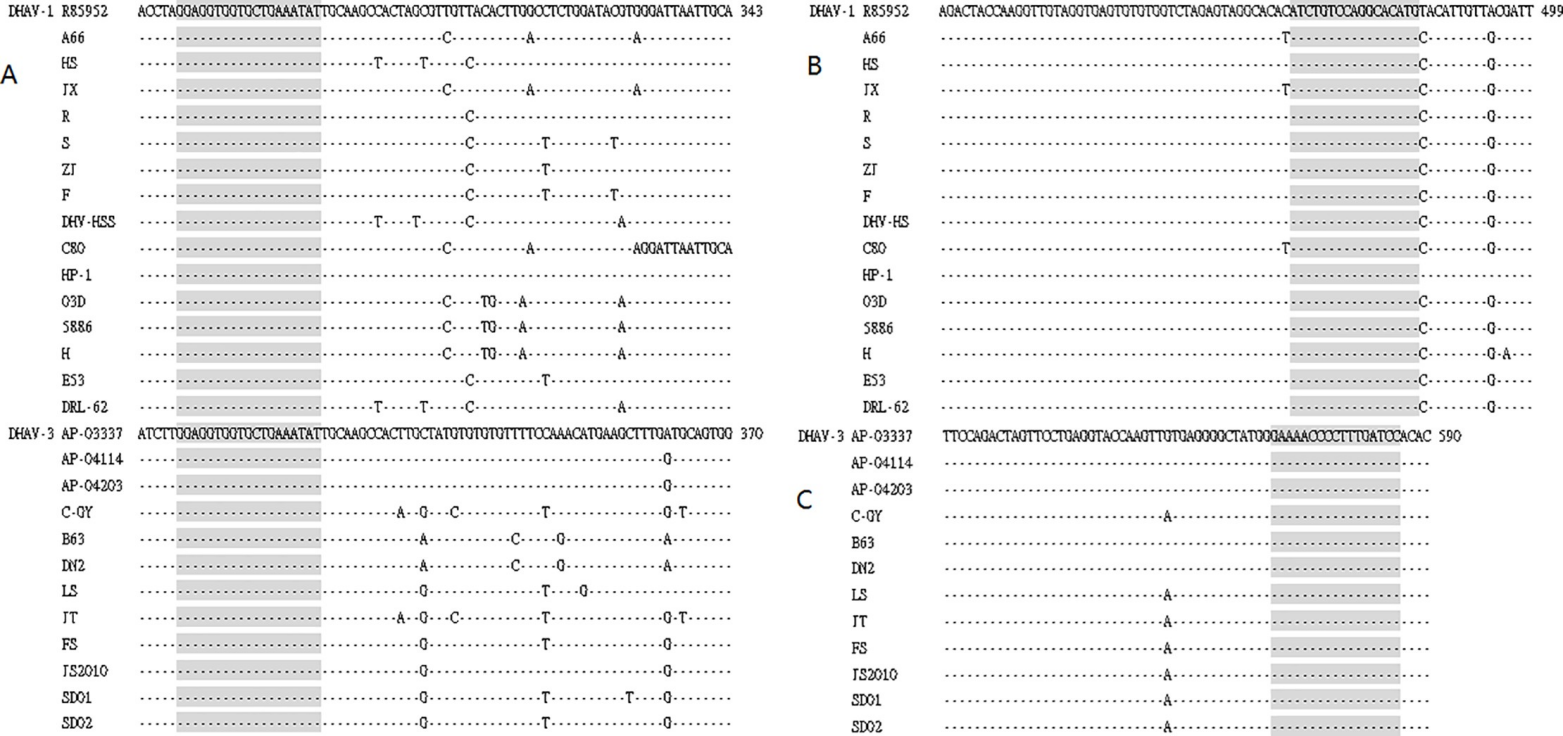
Primers designed by using three alignments of 5’non-conding region sequences from DHAV-1 and DHAV-3 viruses. Identical nucleotides to the top sequences are indicated by dashes. Nucleotide positions are written to the right of sequences. Three conserved regions (in shadow box) 271–289 for DHAV-1 (A) and 298–316 for DHAV-3(A), 468–484 (B) and 570–586 (C) were used for the design of primers for pAF, pA1R, and pA3R, respectively.

Since the 5’-NCR could be used for genotyping analysis, we selected the 5’-NCR for primer design in this study. To locate maximum sequence conservation, the 5’-NCR nucleotide sequences of 16 DHAV (listed in Table 1) from two different genotypes were aligned. Though DHAV-1 and DHAV-3 are different subtypes, the 5’-NCRs of these strains exhibited some regions of conservation in their nucleotide sequences. After careful analysis of the sequences of the 5’-NCR, one absolutely conserved region for both DHAV-1 (nucleotide from 271 to 289) and DHAV-3 (nucleotide from 298 to 316) was selected as PCR common forward primer pAF:5-GGAGGTGGTGCTGAAATAT-3 (Fig 2A). The pAF sequence is completely conserved when it is retrieved (blasted) for similarity to other DHAV-1 and DHAV-3 strains in GenBank (http://www.ncbi.nlm.nih.gov) but without any similarities to DHAV-2 strains, suggesting that pAF is suitable for PCR detection of DHAV-1 and DHAV-3 but not for DHAV-2. To allow the specific detection of DHAV-1 or DHAV-3, reverse primers were designed separately. The criterion of type-specific primers should not only maintain maximum sequence differences between DHAV-1 and DHAV-3 but should also be completely conserved within the same subtype. To find the primer sequence region highly conserved within the same subtype but highly divergent between different subtypes, the 5’-NCR sequences of two different subtypes were aligned separately. The highly conserved region from 468 to 484 was selected as the DHAV-1-specific PCR reverse primer (pA1R) region (Fig 2B) and 570 to 586 was selected as the DHAV-3-specific PCR reverse primer (pA3R) region (Fig 2C). Since DHAV-2 is unavailable in China, the primers designed in this study were mainly intended for DHAV-1 and DHAV-3 detection. Primer information is listed in Table 2. The pAF, pA1R, and pA3R sequences are completely conserved when retrieved for similarity to corresponding DHAV-1 and DHAV-3 strains in GenBank (http://www.ncbi.nlm.nih.gov) but without any similarities to DHAV-2 or other organism, suggesting that pAF, pA1R, and pA3R should be valid for PCR detection of DHAV-1 and DHAV-3 but not for DHAV-2.

**Table 2:** Primer information.

Viruses	Primers	Primer sequences	5’ NCR region	Amplicon size (bp)
DHAV-1	pAF	5-GGAGGTGGTGCTGAAATAT-3	271–289	214
	pA1R	5-CATGTGCCTGGACAGAT-3	468–484	
DHAV-3	pAF	5-GGAGGTGGTGCTGAAATAT-3	298–316	289
	pA3R	5-GGATCAAAGGGGTTTTC-3	570–586	

### Detection of reference DHAV-1 and DHAV-3

**Fig 3:**
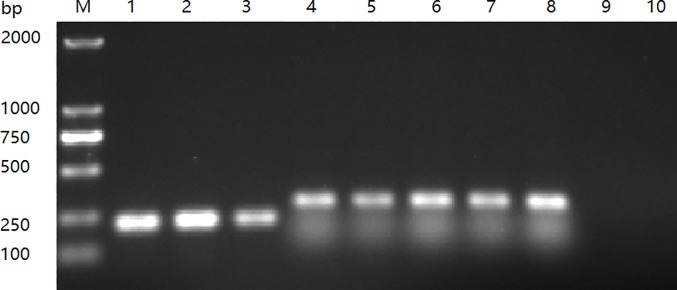
One-tube PCR for differentiating DHAV-1 and DHAV-3. The one-tube PCR was performed using mixed primers. Lanes 1, 2, and 3, DHAV-1 (DRL-62, R85952, and HP-1 strain) PCR products (214 bp), respectively; lanes 4, 5, 6, 7, and 8, DHAV-3 (JT, GY, HLJ-1, ZJ-01309, and SD0517 strain) PCR products (289 bp); lanes 9 and 10, PBS infected allantoic fluids as negative controls. Lane M, DNA molecular marker.

One-tube/one-step RT-PCR was carried out using RNA from different strains (three DHAV-1 viruses, DRL-62, R85952, and HP-1, and five DHAV-3 viruses, JT, GY, HLJ-1, ZJ-01309, and SD0517) as templates. By using three primers (pAF/pA1R/pA3R) in one tube, one-step RT-PCR detected only single products of either 214 bp or 289 bp (Fig 3). The amplified PCR products were then cloned into the pMD18-T vector. The positive clones were purified and submitted for sequencing. The nucleotide sequences of each clone corresponding to the viral RNA in the test tube are exactly the same as the 5’-NCR sequences of the corresponding reference strains in GenBank (data not shown). The cloned amplicon sizes of 214 bp or 289 bp are completely in accordance with the corresponding DHAV-1- or DHAV-3-related subtypes.

### Sensitivity of the one-tube RT-PCR

**Fig 4:**
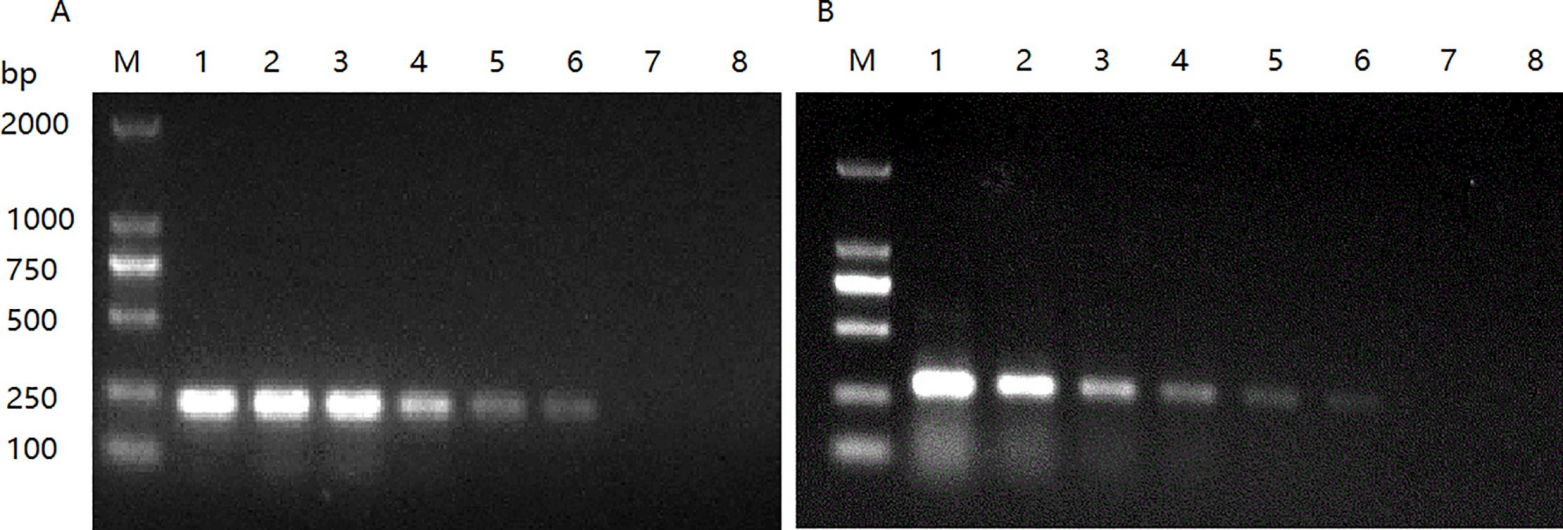
Determination of one-tube RT-PCR detection sensitivity of DHAV-1 (A) and DHAV-3 (B). Lanes 1 to 7, one-tube RT-PCR products obtained from 10^−1^ to 10^−7^ DHAV-1(A) or DHAV-3 (B) virus stock dilutions, respectively; lane 8, PBS infected allantoic fluids as negative controls; M, DNA molecular marker. (A) and (B) representative amplification of RNA templates extracted from serial 10-fold dilutions of DHAV-1 HP-1 and DHAV-3 JT virus stocks, respectively. The lowest dilution of DHAV-1 or DHAV-3 virus stocks detected was the 10^−6^.

Sensitivities of the one-tube RT-PCR for detection representative DHAV-1 (HP-1) and DHAV-3 (JT) were measured by using 10-fold serial dilutions (from 10^−1^ to 10^−7^) of the virus stocks as described previously [27], representing 10^3^ to 10^−3^ ng virion RNA in the reaction at each dilution. In this assay, 10^−6^ dilutions of viruses, equivalent to approximately 10 pg of viral RNA per reaction, were detected (Fig 4). Similar sensitivity was obtained from mixed DHAV-1 and DHAV-3 stock (data not shown).

### Specificity of one-tube RT-PCR

To test the specificity of the designed primers for DHAV-1 and DHAV-3, RNA was extracted from other avian viral isolates corresponding to a number of distinct virus families, including avian influenza viruses (AIV), Newcastle disease virus (NDV), Muscovy duck reovirus (DRV), and duck Tembusu virus (DTMUV). The amplification process was performed with RNA as described above. In no case did the three primers amplify sequence fragments of the appropriate sizes of 214 bp or/and 289 bp (data not shown). Therefore, the primers designed in this study possess a high degree of specificity for the detection and genotyping of DHAV-1 and DHAV-3.

### Detection and genotyping of DHAV-1/DHAV-3 from infected birds

**Fig 5:**
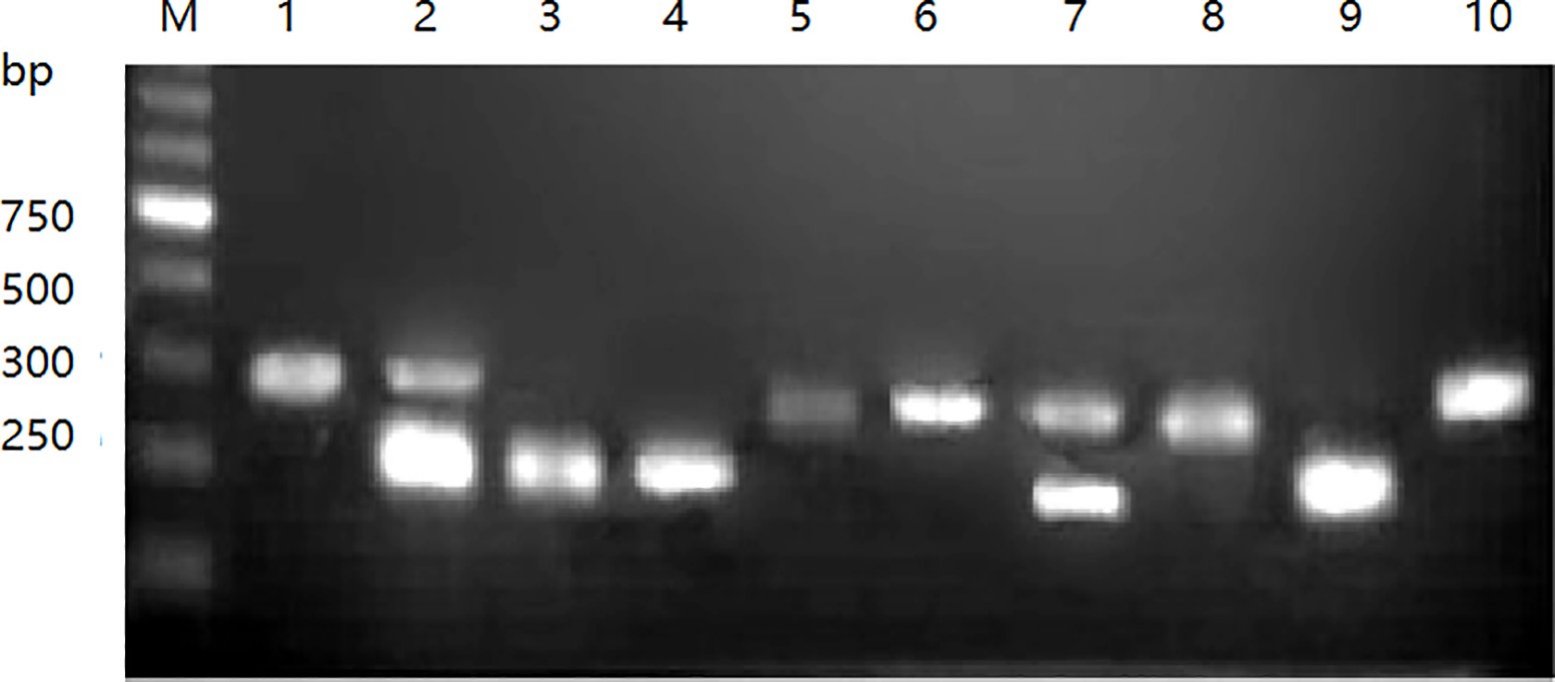
One-tube RT-PCR for genotyping DHAV-1 and DHAV-3 in experimentally infected birds. Liver samples were collected from dead birds after single DHAV-1- or DHAV-3-infection or DHAV-1- and DHAV-3-co-infections. Lanes M, 100 bp DNA marker. Lanes 1, 5, 6, and 10, amplicons (289 bp) from birds with single DHAV-3 infection (represented by JT, GY, ZJ-01309, SD0517, and HLJ-1 strains, respectively) infected birds. Lanes 3, 4, and 9, amplicons (214 bp) from birds with single DHAV-1 infection (represented by DRL-62, R85952, and HP-1). Lanes 2 and 7, amplicons (214 bp and 289 bp) from birds with DHAV-1 and DHAV-3 co-infections [represented by stocks A (mixed HP-1 and JT) and B (mixed DRL-62 and YG), respectively]. No PCR products were detected from PBS control birds (data not shown).

One-day-old SPF ducklings with single DHAV-1 (DRL-62, R85952, and HP-1) or single DHAV-3 (JT, GY, HLJ-1, ZJ-01309, and SD0517) or mixed DHAV-1 and DHAV-3 stocks A (HP-1 and JT) and B (DRL-62 and GY) infections showed 100% (infection with single DRL-62 or R85952 or HP-1), 70% (infection with single JT or GY), 80% (infection with single HLJ-1 or ZJ-01309 or SD0517), 100% (with mixed HP-1 and JT infection) and 90% (with mixed DRL-62 and GY infection) mortalities within two weeks, respectively. All birds, regardless of whether they were infected with single DHAV-1 or DHAV-3 or mixed stocks, showed similar clinical symptoms and pathological changes, including typical hepatitis lesions and enlarged liver with hemorrhages. No signs of disease or death occurred in PBS-negative control birds. RNA from liver homogenates of each group was subjected to one-tube/one-step RT-PCR for detection DHAV-1 and DHAV-3 or for VI/NT. The PCR results showed that single 214-bp and 289-bp fragments were amplified correspondingly from birds infected with single DHAV-1- and DHAV-3-related viruses, respectively (Fig 5, lanes 3, 4, and 9 for DHAV-1; lanes 1, 5, 6, 8, and 10 for DHAV-3). The mixed 214-bp and 289-bp fragments were simultaneously amplified from samples of birds infected with mixed DHAV-1 and DHAV-3 stock A (HP-1 and JT) and stock B (DRL-62 and GY). As demonstrated in Fig 5, amplicons of 214 bp and 289 bp were simultaneously detected from samples of coinfected birds (Fig 5, lanes 2 and 7), which are in accordance with stock A or B in the test tube (data not shown). The sequences of amplicons are consistent with GenBank sequences of corresponding reference viruses, suggesting that this one-tube/one-step RT-PCR could simultaneously detect DHAV-1 and DHAV-3 coinfections in liver samples. No amplicons were detected in bird samples from the PBS control group. Virus isolation combined with neutralization test (VI/NT) are considered “gold standard” methods to test single DHAV-1 and DHAV-3 infection. Neutralization test results showed that allantoic fluids corresponding to reference single DHAV-1 and DHAV-3 infected birds were both neutralized homologous antisera (with r≧1) and only, to some extent, in the presence of undiluted heterologous antisera. These data demonstrated that single amplicons of 214 bp or 289 bp from liver samples are correlated with virus isolation and neutralization tests, suggesting that a one-tube RT-PCR system is also suitable for DHAV-1 and DHAV-3 detection in liver samples of experimentally infected birds. The VI/NT is not applicable for birds coinfected with DHAV-1 and DHAV-3, but the sequence results of 214-bp and 289-bp coamplified products were consistent with those of mixed reference viruses in this study, suggesting that this one-tube/one-step RT-PCR system is also suitable for clinical screening with coinfected birds. To help discriminate between two amplicons, a reference 250 bp line should be included in DNA molecular weight marker (amplicon size 214bp <250 bp and 289 bp >250 bp) and 1.5–2.0% agarose gel is recommended in electrophoresis.

### Screen DHAV-1/DHAV-3 field infection

To screen clinical DHAV-1 and DHAV-3 field infections in China, 55 clinical liver samples were analyzed by one-tube RT-PCR assay and VI/NT. Amplicons with sizes of 214 bp or 289 bp were cloned into the pMD18-T vector, and positive clones were submitted for sequencing. Of the 55 samples, 11 amplicons (approximately 20%) with a single size of 214 bp, 25 amplicons (approximately 45%) with a single size of 289 bp, and 5 amplicons (approximately 9%) with both sizes of 214 bp and 289 bp were obtained. Sequence results showed that 11 clinical samples (isolates) are DHAV-1, 25 are DHAV-3, and 5 are both DHAV-1 and DHAV-3. Because some sequences of amplicons showed 100% homology to each other, we selected and submitted 5 different sequences among DHAV-1 or DHAV-3 or coinfected DHAV-1/-3 to GenBank, and GenBank accession numbers are listed in Table 3.

**Table 3:** Virus detection results by one-tube RT-PCR and virus isolation/neutralization test.

Isolate/Sample	Serotype	Size(bp)	Subtype	5’-NCR identity to (%)		GenBank No
				DHAV-1 DHAV-3		
HRB01507	1	214	DHAV-1	95–99	66–69	MK292314
GZ01506	3	289	DHAV-3	66–68	98–100	MK292305
LY01602A/B	1/3	214/289	DHAV-1/-3	96-100/65-67	66-69/97-99	MK292317/MK292308
FJ01501	3	289	DHAV-3	67–68	97–100	MK292304
BJ01704	3	289	DHAV-3	66–68	97–100	MK292302
JN01706A/B	1/3	214/289	DHAV-1/-3	95-100/66-68	66-69/97-100	MK292316/MK292307
SH01609	3	289	DHAV-3	66–68	97–100	MK292309
CC01508	1	214	DHAV-1	96–100	67–69	MK292312
CF01701A/B	1/3	214/289	DHAV-1/-3	95-100/67-69	66-68/97-100	MK292314
SY01701	1	214	DHAV-1	95–99	65–68	MK292319
JL0153A/B	1/3	214/289	DHAV-1/-3	95-100/66-67	66-68/96-100	MK292315/MK292306
ZZ01507A/B	1/3	214/289	DHAV-1/-3	96-100/66-68	66-69/98-100	MK292321/MK292311
RZ015010	1	214	DHAV-1	95–100	66–68	MK292318
ZH01605	3	289	DHAV-3	66–68	98–100	MK292310
YY01607	1	214	DHAV-1	96–100	66–69	MK292320

## Discussion

The similar phylogenetic topology of the 5′-NCR and polyprotein-encoding gene of reference DHAVs confirmed that the 5′-NCR might be suitable for genetic subtype analysis. The criterion for routine primer design is that a set of primers should accommodate both the similarities and differences exhibited by most of the virus sequences. The completely conserved 5′-GGAGGTGGTGCTGAAATAT-3′ sequence [retrieved from GenBank (http://www.ncbi.nlm.nih.gov)] between DHAV-1 and DHAV-3 might be functionally related and suitable as a universal primer to enable the detection of both serotypes. The 34.3–48.6% diversities in the 5′-NCR sequences suggest that DHAV-1 and DHAV-3 have been evolving separately for a long time, resulting in two distinct genogroups, making this sequence suitable for the design of type-specific primers. Only the main 214-bp or 289-bp product was obtained when nucleic acids of the specified DHAV-1 or DHAV-3 strains were used as the template. With this one-tube RT-PCR, we successfully detected and genotyped samples from dead birds with a single virus infection or a coinfection, validating the approach used in this study. Compared to other molecular genotype methods [8, 10, 19–24], which require amplicon purification, sequencing, and sequencing results analysis, our one-tube system will save at least 6 to 24 h. Similar to other methods, the 10 pg of viral RNA detection was sensitive enough for virus detection and genotyping. The specificity and sensitivity of the one-tube RT-PCR assay were confirmed by VI/NT, and the lack of cross-reaction between DHAV-1 and DHAV-3 also supports the utility of the assay for the detection of DHAV-1 and DHAV-3 coinfection.

The applicability of one-tube RT-PCR to detecting and genotyping clinical samples was also tested. Of the 55 clinical samples tested, 20% were detected as a DHAV-1 infection, 45% as a DHAV-3 infection, and 9% as coinfections, indicating that the DHAV-3 subtype was the main problem facing duck industries in China. The development of a DHAV-3-specific vaccine is therefore highly important, given the current high prevalence of this subtype in duck flocks and the unavailability of measures to control the virus.

This one-tube RT-PCR approach has the following advantages: the size difference of the amplicons is large enough to allow easy interpretation of the results by untrained staff without requiring amplicon sequencing. Moreover, the technique uses fewer costly PCR reagents, and combining the RT and PCR processes into a single step considerably reduces the time, labor, and contamination potential. In addition, this one-step RT-PCR protocol is easy to carry out, even for untrained staff, and it does not yield a separate volume of cDNA as in the two-step and real-time protocols that need to be used in multiple PCRs for different targets. Considering the disease severity and emergence of new serotype DHAV-3, this novel approach would be most helpful to specifically and accurately diagnose the closely related DHAV-1 and DHAV-3 strains and enable their early detection in clinical samples during routine examination. This simple RT-PCR system could be applied in resource-limited settings or on farms for surveillance or for routine epidemiological screening.
